# Interactive Medical Image Segmentation Using Deep Learning With Image-Specific Fine Tuning

**DOI:** 10.1109/TMI.2018.2791721

**Published:** 2018-01-26

**Authors:** Guotai Wang, Wenqi Li, Maria A. Zuluaga, Rosalind Pratt, Premal A. Patel, Michael Aertsen, Tom Doel, Anna L. David, Jan Deprest, Sébastien Ourselin, Tom Vercauteren

**Affiliations:** 1 Wellcome EPSRC Centre for Interventional and Surgical SciencesDepartment of Medical Physics and Biomedical EngineeringUniversity College LondonLondonWC1E 6BTU.K.; 2 Department of Medical Physics and Biomedical EngineeringUniversity College LondonLondonWC1E 6BTU.K.; 3 Facultad de MedicinaUniversidad Nacional de ColombiaBogotá111321Colombia; 4 Amadeus S.A.S.06560Sophia-AntipolisFrance; 5 Department of RadiologyUniversity Hospitals KU Leuven3000LeuvenBelgium; 6 Wellcome EPSRC Centre for Interventional and Surgical SciencesInstitute for Women’s Health, University College LondonLondonWC1E 6BTU.K.; 7 Department of Obstetrics and GynaecologyKU Leuven3000LeuvenBelgium; 8 KU Leuven3000LeuvenBelgium

**Keywords:** Interactive image segmentation, convolutional neural network, fine-tuning, fetal MRI, brain tumor

## Abstract

Convolutional neural networks (CNNs) have achieved state-of-the-art performance for automatic medical image segmentation. However, they have not demonstrated sufficiently accurate and robust results for clinical use. In addition, they are limited by the lack of image-specific adaptation and the lack of generalizability to previously unseen object classes (a.k.a. zero-shot learning). To address these problems, we propose a novel deep learning-based interactive segmentation framework by incorporating CNNs into a bounding box and scribble-based segmentation pipeline. We propose image-specific fine tuning to make a CNN model adaptive to a specific test image, which can be either unsupervised (without additional user interactions) or supervised (with additional scribbles). We also propose a weighted loss function considering network and interaction-based uncertainty for the fine tuning. We applied this framework to two applications: 2-D segmentation of multiple organs from fetal magnetic resonance (MR) slices, where only two types of these organs were annotated for training and 3-D segmentation of brain tumor core (excluding edema) and whole brain tumor (including edema) from different MR sequences, where only the tumor core in one MR sequence was annotated for training. Experimental results show that: 1) our model is more robust to segment previously unseen objects than state-of-the-art CNNs; 2) image-specific fine tuning with the proposed weighted loss function significantly improves segmentation accuracy; and 3) our method leads to accurate results with fewer user interactions and less user time than traditional interactive segmentation methods.

## I. Introduction

Deep learning with convolutional neural networks (CNNs) has achieved state-of-the-art performance for automated medical image segmentation [1]. However, automatic segmentation methods have not demonstrated sufficiently accurate and robust results for clinical use due to the inherent challenges of medical images, such as poor image quality, different imaging and segmentation protocols, and variations among patients [2]. Alternatively, interactive segmentation methods are widely adopted, as they integrate the user’s knowledge and take into account the application requirements for more robust segmentation performance [2]. As such, interactive segmentation remains the state of the art for existing commercial surgical planning and navigation products. Though leveraging user interactions often leads to more robust segmentations, an interactive method should require as short user time as possible to reduce the burden on users. Motivated by these observations, we investigate combining CNNs with user interactions for medical image segmentation to achieve higher segmentation accuracy and robustness with fewer user interactions and less user time. However, there are very few studies on using CNNs for interactive segmentation [3]–[5]. This is mainly due to the requirement of large amounts of annotated images for training, the lack of image-specific adaptation and the demanding balance among model complexity, inference time and memory space efficiency.

The first challenge of using CNNs for interactive segmentation is that current CNNs do not generalize well to previously unseen object classes that are not present in the training set. As a result, they require labeled instances of each object class to be present in the training set. For medical images, annotations are often expensive to acquire as both expertise and time are needed to produce accurate annotations. This limits the performance of CNNs to segment objects for which annotations are not available in the training stage.

Second, interactive segmentation often requires image-specific learning to deal with large context variations among different images, but current CNNs are not adaptive to different test images, as parameters of the model are learned from training images and then fixed in the testing stage, without image-specific adaptation. It has been shown that image-specific adaptation of a pre-trained Gaussian Mixture Model (GMM) helps to improve segmentation accuracy [6]. However, transitioning from simple GMMs to powerful but complex CNNs in this context has not yet been demonstrated.

Third, fast inference and memory efficiency are demanded for interactive segmentation. They can be relatively easily achieved for 2D images, but become much more problematic for 3D images. For example, DeepMedic [7] works on 3D local patches to reduce memory requirements but results in a slow inference. HighRes3DNet [8] works on 3D whole images with relatively fast inference but needs a large amount of GPU memory, leading to high hardware requirements. To make a CNN-based interactive segmentation method efficient to use, enabling CNNs to respond quickly to user interactions and to work on a machine with limited GPU resources (e.g., a standard desktop PC or a laptop) is desirable. DeepIGeoS [5] combines CNNs with user interactions and has demonstrated good interactivity. However, it has a lack of adaptability to unseen image contexts.

This paper presents a new framework to address these challenges for deep learning-based interactive segmentation. To generalize to previously unseen objects, we propose a bounding-box-based segmentation pipeline that extracts the foreground from a given region of interest, and design a 2D and a 3D CNN with good compactness to avoid over-fitting. To make CNNs adaptive to different test images, we propose image-specific fine-tuning. In addition, our networks consider a balance among receptive field, inference time and memory efficiency so as to be responsive to user interactions and have low requirements in terms of GPU resources.

### A. Contributions

The contributions of this work are four-fold. First, we propose a novel deep learning-based framework for interactive 2D and 3D medical image segmentation by incorporating CNNs into a bounding box and scribble-based binary segmentation pipeline. Second, we propose image-specific fine-tuning to adapt a CNN model to each test image independently. The fine-tuning can be either unsupervised (without additional user interactions) or supervised by user-provided scribbles. Third, we propose a weighted loss function considering network and interaction-based uncertainty during the image-specific fine-tuning. Fourth, we present the first attempt to employ CNNs to deal with previously unseen objects (a.k.a. zero-shot learning) in the context of image segmentation. The proposed framework does not require all the object classes to be annotated for training. Thus, it can be applied to new organs or new segmentation protocols directly.

### B. Related Works

#### 1) CNNs for Image Segmentation:

For natural image segmentation, FCN [9] and DeepLab [10] are among the state-of-the-art performing methods. For 2D biomedical image segmentation, efficient networks such as U-Net [11], DCAN [12] and Nabla-net [13] have been proposed. For 3D volumes, patch-based CNNs have been proposed for segmentation of the brain tumor [7] and pancreas [14], and more powerful end-to-end 3D CNNs include V-Net [15], HighRes3DNet [8], and 3D deeply supervised network [16].

#### 2) Interactive Segmentation Methods:

A wide range of interactive segmentation methods have been proposed [2]. Representative methods include Graph Cuts [17], Random Walks [18] and GeoS [19]. Machine learning has been popularly used to achieve high accuracy and interaction efficiency. For example, GMMs are used by GrabCut [20] to segment color images. Online Random Forests (ORFs) are employed by Slic-Seg [21] for placenta segmentation from fetal Magnetic Resonance images (MRI). In [22], active learning is used to segment 3D Computed Tomography (CT) images. They have achieved more accurate segmentations with fewer user interactions than traditional interactive segmentation methods.

To combine user interactions with CNNs, DeepCut [3] and ScribbleSup [23] propose to leverage user-provided bounding boxes or scribbles, but they employ user interactions as sparse annotations for the training set rather than as guidance for dealing with test images. 3D U-Net [24] learns from annotations of some slices in a volume and produces a dense 3D segmentation, but is not responsive to user interactions. In [4], an FCN is combined with user interactions for 2D RGB image segmentation, without adaptation for medical images. DeepIGeoS [5] uses geodesic distance transforms of scribbles as additional channels of CNNs for interactive segmentation, but cannot deal with previously unseen object classes.

#### 3) Model Adaptation:

Previous learning-based interactive segmentation methods often employ image-specific models. For example, GrabCut [20] and Slic-Seg [21] learn from the target image with GMMs and ORFs, respectively, so that they can be well adapted to the specific target image. Learning a model from a training set with image-specific adaptation in the testing stage has also been used to improve the segmentation performance. For example, an adaptive GMM has been used to address the distribution mismatch between the training and test images [6]. For CNNs, fine-tuning [25] is used for domain-wise model adaptation to address the distribution mismatch between different training sets. However, to the best of our knowledge, this paper is the first work to propose image-specific model adaptation for CNNs.

## II. Method

**Fig. 1.**
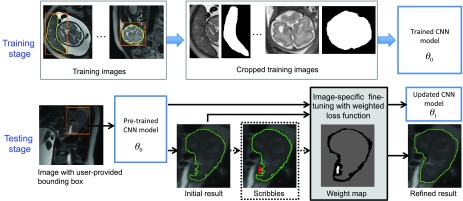
The proposed Bounding box and Image-specific Fine-tuning-based Segmentation (BIFSeg). 2D images are shown as examples. During training, each instance is cropped with its bounding box, and the CNN is trained for binary segmentation. In the testing stage, image-specific fine-tuning with optional scribbles and a weighted loss function is used. Note that the object class (e.g. maternal kidneys) for testing may have not been present in the training set.

The proposed interactive framework with Bounding box and Image-specific Fine-tuning-based Segmentation (BIFSeg) is depicted in Fig. 1. To deal with different (including previously unseen) objects in a unified framework, we propose to use a CNN that takes as input the content of a bounding box of one instance and gives a binary segmentation for that instance. In the testing stage, the user provides a bounding box, and BIFSeg extracts the region inside the bounding box and feeds it into the pre-trained CNN with a forward pass to obtain an initial segmentation. This is based on the fact that our CNNs are designed and trained to learn some common features, such as saliency, contrast and hyper-intensity, across different objects, which helps to generalize to unseen objects. Then we use unsupervised (without additional user interactions) or supervised (with user-provided scribbles) image-specific fine-tuning to further refine the segmentation. This is because there is likely a mismatch between the common features learned from the training set and those in (previously unseen) test objects. Therefore, we use fine-tuning to leverage image-specific features and make our CNNs adaptive to a specific test image for better segmentation. Our framework is general, flexible and can handle both 2D and 3D segmentations with few assumptions of network structures. In this paper, we choose to use the state-of-the-art network structures proposed in [5] for their compactness and efficiency. The contribution of BIFSeg is nonetheless largely different from [5] as BIFSeg focuses on segmentation of previously unseen object classes and fine-tunes the CNN model on the fly for image-wise adaptation that can be guided by user interactions.

### A. CNN Models

**Fig. 2.**
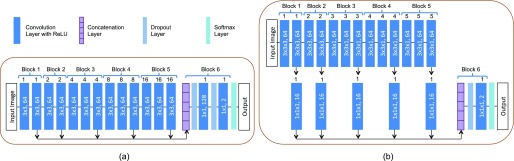
Our resolution-preserving networks with dilated convolution for 2D segmentation (a) and 3D segmentation (b). The numbers in dark blue boxes denote convolution kernel sizes and numbers of output channels, and the numbers on the top of these boxes denote dilation parameters.

For 2D images, we adopt the P-Net [5] for bounding box-based binary segmentation. The network is resolution-preserving using dilated convolution [10]. As shown in Fig. 2(a), it consists of six blocks with a receptive field of $181\times 181$. The first five blocks have dilation parameters of 1, 2, 4, 8 and 16, respectively, so they capture features at different scales. Features from these five blocks are concatenated and fed into block6 that serves as a classifier. A softmax layer is used to obtain probability-like outputs. In the testing stage, we update the model based on image-specific fine-tuning. To ensure efficient fine-tuning and fast response to user interactions, we only fine-tune parameters of the classifier (block6). Thus, features in the concatenation layer for the test image can be stored before the fine-tuning.

For 3D images, we use a network extended from P-Net, as shown in Fig. 2(b). It considers a trade-off among receptive field, inference time and memory efficiency. The network has an anisotropic receptive field of $85\times 85\times 9$. Compared with slice-based networks, it employs 3D contexts. Compared with large isotropic 3D receptive fields [8], it has less memory consumption [26]. Besides, anisotropic acquisition is often used in Magnetic Resonance (MR) imaging. We use $3\times 3\times 3$ kernels in the first two blocks and $3\times 3\times 1$ kernels in block3 to block5. Similar to P-Net, we fine-tune the classifier (block6) with pre-computed concatenated features. To save space for storing the concatenated features, we use $1\times 1\times 1$ convolutions to compress the features in block1 to block5 and then concatenate them. We refer to this 3D network with feature compression as PC-Net.

### B. Training of CNNs

The training stage for 2D/3D segmentation is shown in the first row of Fig. 1. Consider a $K$-ary segmentation training set $T=\{(X_{1},Y_{1}), (X_{2}, Y_{2}), \ldots \}$ where $X_{p}$ is one training image and $Y_{p}$ is the corresponding label map. The label set of $T$ is $\{0, 1, 2, \ldots, K-1\}$ with 0 being the background label. Let $N_{k}$ denote the number of instances of the $k$th object type, so the total number of instances is $\hat {N} = \sum _{k}{N_{k}}$. Each image $X_{p}$ can have instances of multiple object classes. Suppose the label of the $q$th instance in $X_{p}$ is $l_{pq}$, $Y_{p}$ is converted into a binary image $Y_{pq}$ based on whether the value of each pixel in $Y_{p}$ equals to $l_{pq}$. The bounding box $B_{pq}$ of that training instance is automatically calculated based on $Y_{pq}$ and expanded by a random margin in the range of 0 to 10 pixels/voxels. $X_{p}$ and $Y_{pq}$ are cropped based on $B_{pq}$. Thus, $T$ is converted into a cropped set $\hat {T}=\{(\hat {X}_{1},\hat {Y}_{1}), (\hat {X}_{2}, \hat {Y}_{2}), \ldots \}$ with size $\hat {N}$ and label set $\{0, 1\}$ where 1 is the label of the instance foreground and 0 the background. With $\hat {T}$, the CNN model (e.g., P-Net or PC-Net) is trained to extract the target from its bounding box, which is a binary segmentation problem irrespective of the object type. A cross entropy loss function is used for training.

### C. Unsupervised and Supervised Image-Specific Fine-Tuning

In the testing stage, let $\hat {X}$ denote the sub-image inside a user-provided bounding box and $\hat {Y}$ be the target label of $\hat {X}$. The set of parameters of the trained CNN is $\theta $. With the initial segmentation $\hat {Y}_{0}$ obtained by the trained CNN, the user may provide (i.e., supervised) or not provide (i.e., unsupervised) a set of scribbles to guide the update of $\hat {Y}_{0}$. Let $S^{f}$ and $S^{b}$ denote the scribbles for foreground and background, respectively, so the entire set of scribbles is $S = S^{f} \cup S^{b}$. Let $s_{i}$ denote the user-provided label of a pixel in the scribbles, then we have $s_{i}=1$ if $i\in S_{f}$ and $s_{i}=0$ if $i\in S_{b}$. We minimize an objective function that is similar to GrabCut [20] but we use P-Net or PC-Net instead of a GMM:

\begin{align*}&\underset {\hat {Y}, \theta }{\text {arg min}}~\left \{{ E(\hat {Y},\theta) = \sum _{i}\phi (\hat {y}_{i}|\hat {X},\theta) + \lambda \sum _{i,j}\psi (\hat {y}_{i}, \hat {y}_{j}|\hat {X})}\right \} \notag \\&\text {subject to}:~\hat {y}_{i} = s_{i} \quad \text {if } i \in S \end{align*}

where $E(\hat {Y},\theta)$ is constrained by user interactions if $S$ is not empty. $\phi $ and $\psi $ are the unary and pairwise energy terms, respectively. $\lambda $ is the weight of $\psi $. An unconstrained optimization of an energy similar to $E$ was used in [3] for weakly supervised learning. In that work, the energy was based on the probability and label map of all the images in a training set, which was a different task from ours, as we focus on a single test image. We follow a typical choice of $\psi $ [17]:

\begin{equation*} \psi (\hat {y}_{i}, \hat {y}_{j}|\hat {X})= [\hat {y}_{i} \neq \hat {y}_{j}] \text {exp}\left ({-\frac {(\hat {X}(i)-\hat {X}(j))^{2}}{2\sigma ^{2}}}\right)\cdot \frac {1}{d_{ij}} \quad \end{equation*}

where $[\cdot]$ is 1 if $\hat {y}_{i} \neq \hat {y}_{j}$ and 0 otherwise. $d_{ij}$ is the Euclidean distance between pixel $i$ and pixel $j$. $\sigma $ controls the effect of intensity difference. $\phi $ is defined as:

\begin{align*} \phi (\hat {y}_{i}|\hat {X},\theta)=&-\text {log}P(\hat {y}_{i}|\hat {X},\theta) \notag \\=&-\Big (\hat {y}_{i}\text {log} p_{i} + (1-\hat {y}_{i})\text {log} (1 - p_{i})\Big) \end{align*}

where $P(\hat {y}_{i}|\hat {X},\theta)$ is the probability given by softmax output of the CNN, and $p_{i}=P(\hat {y}_{i} = 1|\hat {X},\theta)$ is the probability of pixel $i$ belonging to the foreground.

The optimization of Eq. (1) can be decomposed into steps that alternatively update the segmentation label $\hat {Y}$ and network parameters $\theta $ [3], [20]. In the label update step, we fix $\theta $ and solve for $\hat {Y}$, and Eq. (1) becomes a Conditional Random Field (CRF) problem:

\begin{align*}&\underset {\hat {Y}}{\text {arg min}}~\left \{{ E(\theta) = \sum _{i}\phi (\hat {y}_{i}|\hat {X},\theta) + \lambda \sum _{i,j}\psi (\hat {y}_{i}, \hat {y}_{j}|\hat {X})}\right \} \notag \\&\text {subject to}:~\hat {y}_{i} = s_{i} \quad \text {if } i \in S \end{align*}

For implementation ease, the constrained optimization in Eq. (4) is converted to an unconstrained equivalent:

\begin{align*}&\hspace {-5pc}\underset {\hat {Y}}{\text {arg min}}~\left \{{ \sum _{i}\phi '(\hat {y}_{i}|\hat {X},\theta) + \lambda \sum _{i,j}\psi (\hat {y}_{i}, \hat {y}_{j}|\hat {X}) }\right \} \\ \phi '(\hat {y}_{i}|\hat {X},\theta)=&\begin{cases} +\infty & \text {if } i \in S \text {and }\hat {y}_{i}=s_{i}\\ 0 & \text {if } i \in S \text {and }\hat {y}_{i}\neq s_{i}\\ -\text {log} P(\hat {y}_{i}|\hat {X},\theta) & \text {otherwise}\\ \end{cases}\qquad \end{align*}

Since $\theta $ and therefore $\phi '$ are fixed, and $\psi $ is submodular, Eq. (5) can be solved by Graph Cuts [17]. In the network update step, we fix $\hat {Y}$ and solve for $\theta $:

\begin{align*}&\underset {\theta }{\text {arg min}}~\left \{{ E(\hat {Y})=\sum _{i}\phi (\hat {y}_{i}|\hat {X},\theta) }\right \}\notag \\&\text {subject to}:~\hat {y}_{i} = s_{i} \quad \text {if } i \in S \end{align*}

Thanks to the constrained optimization in Eq. (4), the label update step necessarily leads to $\hat {y}_{i} = s_{i}$ for $i\in S$. Eq. (7) can be treated as an unconstrained optimization:

\begin{equation*} \underset {\theta }{\text {arg min}}~\left \{{- \sum _{i}\Big (\hat {y}_{i}\text {log} p_{i} + (1-\hat {y}_{i})\text {log}(1-p_{i})\Big)}\right \} \end{equation*}

### D. Weighted Loss Function During Network Update Step

During the network update step, the CNN is fine-tuned to fit the current segmentation $\hat {Y}$. Differently from a standard learning process that treats all the pixels equally, we propose to weight different kinds of pixels considering their confidence. First, user-provided scribbles have much higher confidence than the other pixels, and they should have a higher impact on the loss function, leading to a weighted version of Eq. (3):

\begin{align*} \phi (\hat {y}_{i}| \hat {X},\theta)=&-w(i)\text {log}P(\hat {y}_{i}|\hat {X},\theta) \\ w(i)=&\begin{cases} \omega & \text {if } i\in S \\ 1 & \text {otherwise}\\ \end{cases} \end{align*}

where $\omega \geq 1$ is the weight associated with scribbles. $\phi $ defined in Eq. (9) allows Eq. (4) to remain unchanged for the label update step. In the network update step, Eq. (8) becomes:

\begin{equation*} \underset {\theta }{\text {arg min}} ~\left \{{\!- \sum _{i}w(i)\Big (\hat {y}_{i}\text {log} p_{i} + (1-\hat {y}_{i})\text {log}(1-p_{i})\Big)\!}\right \} \qquad \end{equation*}

Note that the energy optimization problem of Eq. (1) remains well-posed with Eq. (9), (10), and (11).

**Fig. 3.**
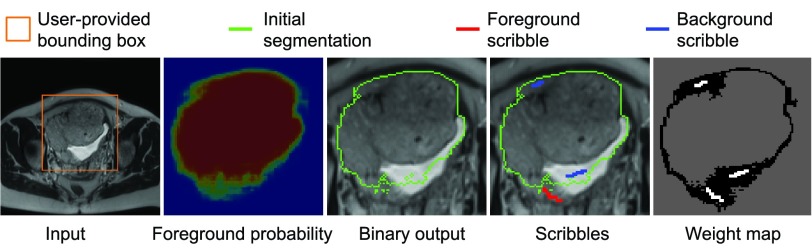
An example of weight map for image-specific fine-tuning. The weight is 0 for pixels with high uncertainty (black), $\omega $ for scribbles (white), and 1 for the remaining pixels (gray).

Second, $\hat {Y}$ may contain mis-classified pixels that can mis-lead the network update process. To address this problem, we propose to fine-tune the network by ignoring pixels with high uncertainty (low confidence) in the test image. We propose to use network-based uncertainty and scribble-based uncertainty. The network-based uncertainty is based on the network’s softmax output. Since $\hat {y}_{i}$ is highly uncertain (has low confidence) if $p_{i}$ is close to 0.5, we define the set of pixels with high network-based uncertainty as $U_{p} = \{i|t_{0} < p_{i} < t_{1}\}$ where $t_{0}$ and $t_{1}$ are the lower and higher threshold values of foreground probability, respectively. The scribble-based uncertainty is based on the geodesic distance to scribbles. Let $G(i, S^{f})$ and $G(i, S^{b})$ denote the geodesic distance [19] from pixel $i$ to $S^{f}$ and $S^{b}$, respectively. Since the scribbles are drawn on mis-segmented areas for refinement, it is likely that pixels close to $S$ have been incorrectly labeled by the initial segmentation. Let $\epsilon $ be a threshold value for the geodesic distance. We define the set of pixels with high scribble-based uncertainty as $U_{s} = U_{s}^{f} \cup U_{s}^{b}$ where $U_{s}^{f} = \{i|i\notin S, G(i, S^{f})<\epsilon, \hat {y_{i}}=0\}$, $U_{s}^{b} = \{i|i\notin S, G(i, S^{b})<\epsilon, \hat {y_{i}}=1\}$. Therefore, a full version of the weight function is (an example is shown in Fig. 3):

\begin{equation*} w(i) = \begin{cases} \omega & \text {if } i\in S \\ 0 & \text {if } i\in U_{p} \cup U_{s} \\ 1 & \text {otherwise}\\ \end{cases} \end{equation*}

The new definition of $w(i)$ is well motivated in the network update step. However, in the label update step, introducing zero unary weights in Eq. (4) would make the label update of corresponding pixels entirely driven by the pairwise potentials. Therefore, we choose to keep Eq. (4) unchanged.

### E. Implementation Details

We used the Caffe^1^ [27] library to implement our P-Net and PC-Net.^2^ The training process was done via one node of the Emerald cluster^3^ with two 8-core E5-2623v3 Intel Haswells, a K80 NVIDIA GPU and 128GB memory. To deal with different organs and different modalities, the region inside a bounding box was normalized by the mean value and standard deviation of that region, and then used as the input of the CNNs. In the training stage, the bounding box was automatically generated based on the ground truth label with a random margin in the range of 0 to 10 pixels/voxels. We used cross entropy loss function and stochastic gradient decent with momentum 0.9, batch size 1, weight decay $5\times 10^{-4}$, maximal number of iterations 80k and initial learning 10^−3^ that was halved every 5k iterations.^1^http://caffe.berkeleyvision.org^2^Code available at: https://cmiclab.cs.ucl.ac.uk/GIFT-Surg/BIFSeg^3^http://www.ses.ac.uk/high-performance-computing/emerald

In the testing stage, the trained CNN models were deployed to a MacBook Pro (OS X 10.9.5) with 16GB RAM, an Intel Core i7 CPU running at 2.5GHz and an NVIDIA GeForce GT 750M GPU. A Matlab GUI and a PyQt GUI were used for user interactions on 2D and 3D images, respectively. For image-specific fine-tuning, $\hat {Y}$ and $\theta $ were alternatively updated for four iterations. In each network update step, we used a learning rate 10^−2^ and iteration number 20. We used a grid search with the training data to get proper values of $\lambda $, $\sigma $, $t_{0}$, $t_{1}$, $\epsilon $ and $\omega $, and fixed them as global parameters during testing. Their numerical values are listed in the specific experimental sections III-B and III-C.

## III. Experiments and Results

We validated the proposed framework with two applications: 2D segmentation of multiple organs from fetal MRI and 3D segmentation of brain tumors from contrast enhanced T1-weighted (T1c) and Fluid-attenuated Inversion Recovery (FLAIR) images. For both applications, we additionally investigated the segmentation performance on previously unseen objects that were not present in the training set.

### A. Comparison Methods and Evaluation Metrics

To investigate the performance of different networks with the same bounding box, we compared P-Net with FCN [9] and U-Net [11] for 2D images, and compared PC-Net with DeepMedic [7] and HighRes3DNet [8] for 3D images.^4^ The original DeepMedic works on multiple modalities, and we adapted it to work on a single modality. All these methods were evaluated on the laptop during the testing except for HighRes3DNet that was run on the cluster due to the laptop’s limited GPU memory. To validate the proposed unsupervised/supervised image-specific fine-tuning, we compared BIFSeg with 1) the initial output of P-Net/ PC-Net, 2) post-processing the initial output with a CRF (using user interactions as hard constraints if they were provided), and 3) image-specific fine-tuning based on Eq. (1) with $w(i) =1 $ for all the pixels, which is referred to as BIFSeg(-w).^4^DeepMedic and HighRes3DNet were implemented in http://niftynet.io

BIFSeg was also compared with other interactive methods: GrabCut [20], Slic-Seg [21] and Random Walks [18] for 2D segmentation, and GeoS [19], GrowCut [28] and 3D GrabCut [29] for 3D segmentation. The 2D/3D GrabCut used the same bounding box as used by BIFSeg, and they used 3 and 5 components for the foreground and background GMMs, respectively. Slic-Seg, Random Walks, GeoS and GrowCut required scribbles without a bounding box for segmentation. The segmentation results by an Obstetrician and a Radiologist were used for evaluation. For each method, each user provided scribbles to update the result multiple times until the user accepted it as the final segmentation. The Dice score between a segmentation and the ground truth was used for quantitative evaluations: $\text {Dice}=2|\mathcal {R}_{a}\cap \mathcal {R}_{b}|/(|\mathcal {R}_{a}|+|\mathcal {R}_{b}|)$ where $\mathcal {R}_{a}$ and $\mathcal {R}_{b}$ denote the region segmented by an algorithm and the ground truth, respectively. We used a paired Student’s $t$-test to determine whether the performance difference between two segmentation methods was significant [30]. The $p$-value, i.e., the probability of achieving a more extreme value than the observed segmentation performance difference, when the null hypothesis is true, was calculated for significance assessment.

### B. 2D Segmentation of Multiple Organs From Fetal MRI

#### 1) Data:

Single-shot Fast Spin Echo (SSFSE) was used to acquire stacks of T2-weighted MR images from 18 patients with pixel size 0.74 to 1.58 mm and inter-slice spacing 3 to 4 mm. Due to the large inter-slice spacing and inter-slice motion, interactive 2D segmentation is more suitable than direct 3D segmentation [21]. We performed data splitting at patient level and used images from 10, 2, 6 patients for training, validation and testing, respectively. The training set consisted of 333 and 213 2D instances of the placenta and fetal brain, respectively. The validation set contained 70, 25, 36 and 41 2D instances of the placenta, fetal brain, fetal lungs and maternal kidneys, respectively. The testing set consisted of 165, 80, 114 and 124 2D instances of the placenta, fetal brain, fetal lungs and maternal kidneys, respectively. Here the fetal brain and the placenta were previously seen objects, and the fetal lungs and maternal kidneys were previously unseen objects. Manual segmentations by a Radiologist were used as the ground truth. The P-Net was used for this segmentation task. The bounding boxes of organs in the training set had an original side length of 98±59 pixels. To deal with organs at different scales, we resized the input of P-Net so that the minimal value of width and height was 96 pixels. In the testing stage, the output of BIFSeg for one object was resized to fit its bounding box in the original image. Parameter setting was $\lambda = 3.0$, $\sigma = 0.1$, $t_{0} = 0.2$, $t_{1} = 0.7$, $\epsilon = 0.2$, $\omega = 5.0$ based on a grid search with the training data (i.e., fetal lungs and maternal kidneys were not used for parameter learning).

#### 2) Initial Segmentation Based on P-Net:

**Fig. 4.**
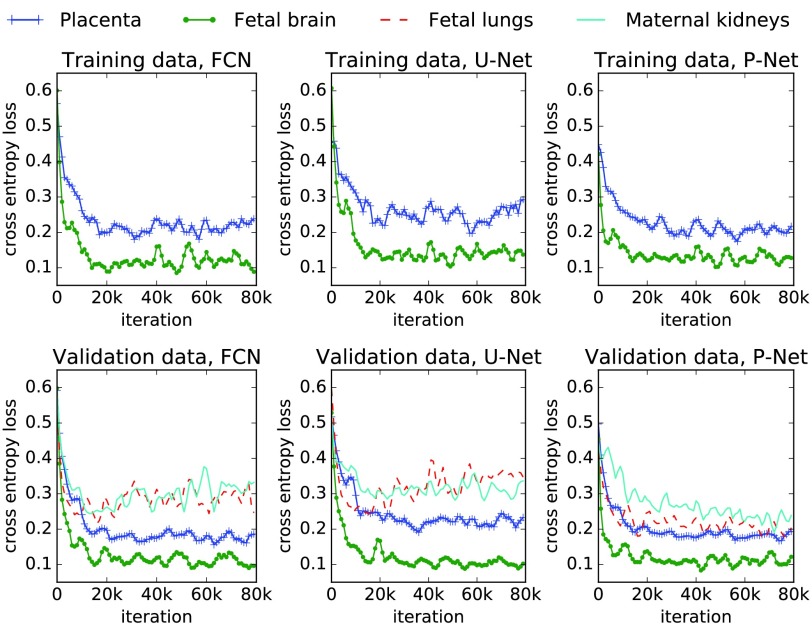
Evolution of cross entropy loss on training and validation data during the training stage of different networks for 2D fetal MRI segmentation. Fetal lungs and maternal kidneys were not present in the training set.

**Fig. 5.**
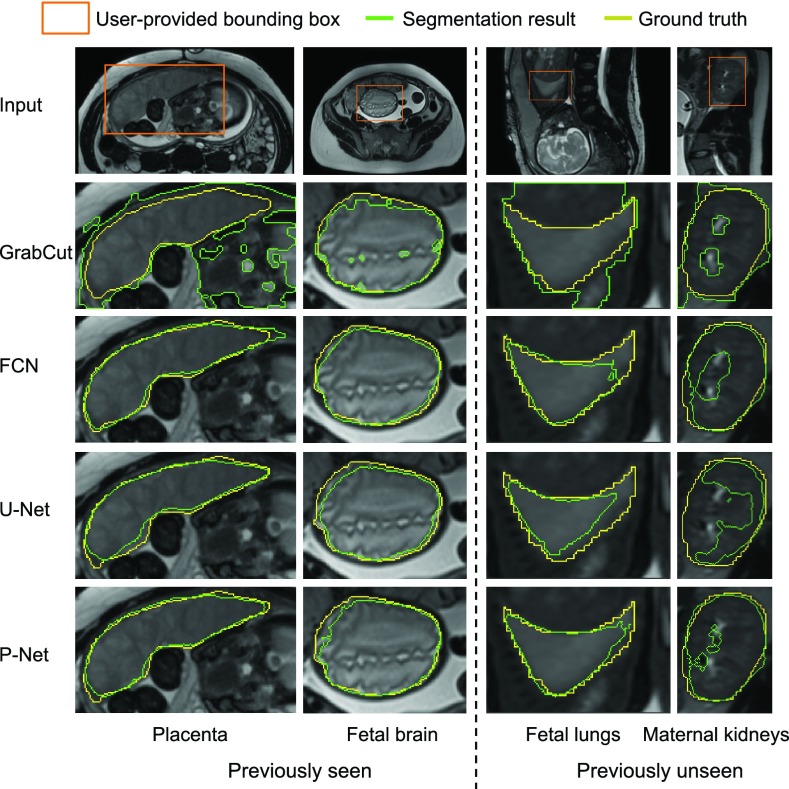
Visual comparison of initial segmentation of multiple organs from fetal MRI with a bounding box. All the methods use the same bounding box for each test instance. Note that fetal lungs and maternal kidneys are previously unseen objects but P-Net works well on them.

Fig. 4 presents the evolution of the loss on the training and validation data with FCN, U-Net and P-Net during the training stage. It shows that FCN and U-Net tend to over-fit the placenta and fetal brain in the training set, while P-Net generalizes better to previously unseen fetal lungs and maternal kidneys in comparison. Fig. 5 shows the initial segmentation of different organs from fetal MRI with user-provided bounding boxes. It can be observed that GrabCut achieves a poor segmentation except for the fetal brain where there is a good contrast between the target and the background. For the placenta and fetal brain, FCN, U-Net and P-Net achieve visually similar results that are close to the ground truth. However, for fetal lungs and maternal kidneys that are previously unseen in the training set, FCN and U-Net lead to a large region of under-segmentation. In contrast, P-Net performs noticeably better than FCN and U-Net when dealing with these two unseen objects. A quantitative evaluation of these methods is listed in Table I. It shows that P-Net achieves the best accuracy for unseen fetal lungs and maternal kidneys with average machine time 0.16s.

**TABLE I:** Quantitative Comparison of Initial Fetal MRI Segmentation From a Bounding Box. ${T}_{m}$ is the Machine Time. ${\wedge} $ Denotes Previously Unseen Objects. In Each Row, Bold Font Denotes the Best Value. * Denotes ${p}$-Value < 0.05 Compared With the Others

		FCN	U-Net	P-Net	GrabCut
Dice(%)	P	85.31±8.73	82.86±9.85	84.57±8.37	62.90±12.79
	FB	89.53±3.91	89.19±5.09	89.44±6.45	83.86±14.33
	FL$^{\wedge}$	81.68±5.95	80.64±6.10	83.59±6.42*	63.99±15.86
	MK$^{\wedge}$	83.58±5.48	75.20±11.23	85.29±5.08*	73.85±7.77
$T_{m}$(s)		0.11±0.04*	0.24±0.07	0.16±0.05	1.62±0.42

P: Placenta, FB: Fetal brain, FL: Fetal lungs, MK: Maternal kidneys.

#### 3) Unsupervised Image-Specific Fine-Tuning:

**Fig. 6.**
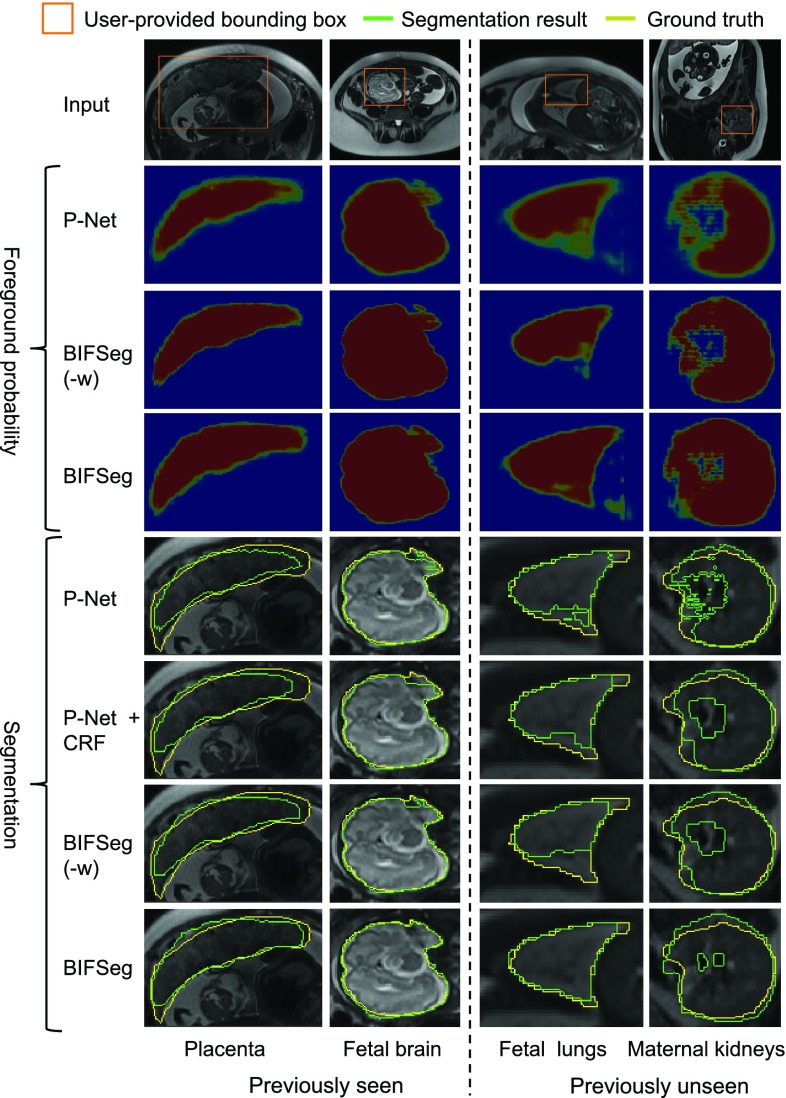
Visual comparison of P-Net and three unsupervised refinement methods for fetal MRI segmentation. The foreground probability is visualized by heatmap.

For unsupervised refinement, the initial segmentation obtained by P-Net was refined by CRF, BIFSeg(-w) and BIFSeg without additional scribbles, respectively. The results are shown in Fig. 6. The second to fourth rows show the foreground probability obtained by P-Net before and after the fine-tuning. In the second row, the initial output of P-Net has a probability around 0.5 for many pixels, which indicates a high uncertainty. After image-specific fine-tuning, most pixels in the outputs of BIFSeg(-w) and BIFSeg have a probability close to 0.0 or 1.0. The remaining rows show the outputs of P-Net and the three refinement methods, respectively. The visual comparison shows that BIFSeg performs better than P-Net + CRF and BIFSeg(-w). Quantitative measurements are presented in Table II. It shows that BIFSeg achieves a larger improvement of accuracy from the initial segmentation when compared with the use of CRF or BIFSeg(-w). In this 2D case, BIFSeg takes 0.72s in average for unsupervised image-specific fine-tuning.

**TABLE II:** Quantitative Comparison of P-Net and Three Unsupervised Refinement Methods for Fetal MRI Segmentation. ${T}_{m}$ is the Machine Time for Refinement. ${\wedge} $ Denotes Previously Unseen Objects. In Each Row, Bold Font Denotes the Best Value. * Denotes ${p}$-Value < 0.05 Compared With the Others

		P-Net	P-Net+CRF	BIFSeg(-w)	BIFSeg
Dice(%)	P	84.57±8.37	84.87±8.14	82.74±10.91	86.41±7.50*
	FB	89.44±6.45	89.55±6.52	89.09±8.08	90.39±6.44
	FL$^{\wedge}$	83.59±6.42	83.87±6.52	82.17±8.87	85.35±5.88*
	MK$^{\wedge}$	85.29±5.08	85.45±5.21	84.61±6.21	86.33±4.28*
$T_{m}$ (s)		-	0.02±0.01*	0.71±0.12	0.72±0.12

P: Placenta, FB: Fetal brain, FL: Fetal lungs, MK: Maternal kidneys.

#### 4) Supervised Image-Specific Fine-Tuning:

**Fig. 7.**
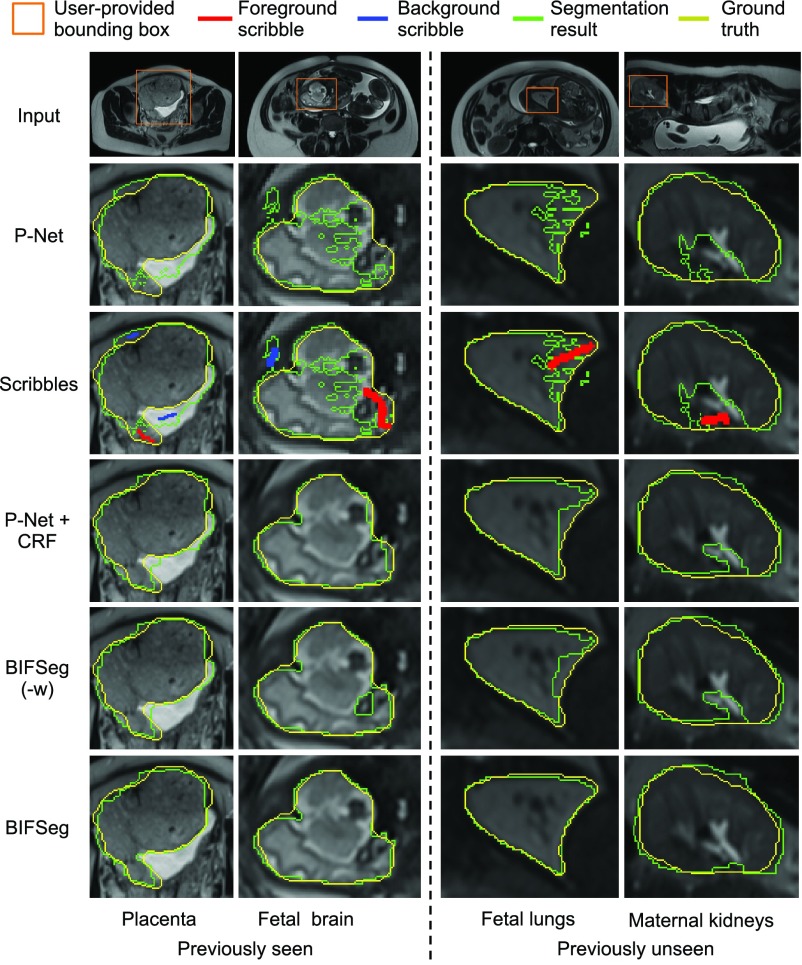
Visual comparison of P-Net and three supervised refinement methods for fetal MRI segmentation. The same initial segmentation and scribbles are used for P-Net + CRF, BIFSeg(-w) and BIFSeg.

**Fig. 8.**
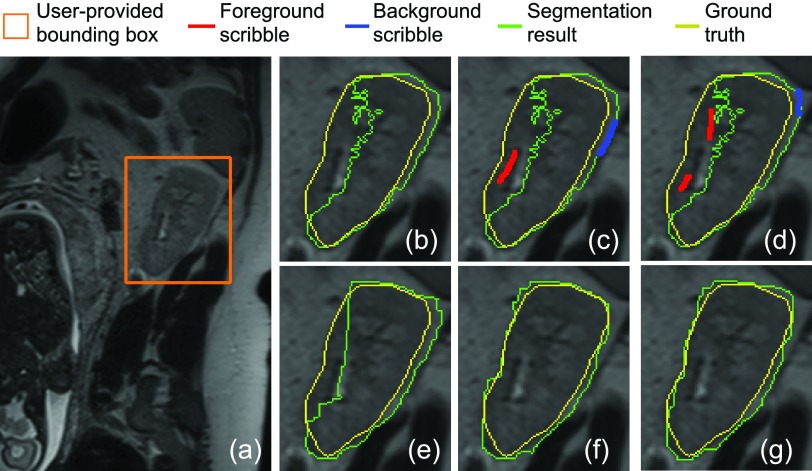
Unsupervised and supervised fine-tuning results of BIFSeg for the same instance of previously unseen maternal kidneys. (a) shows the user-provided bounding box. (b) is the initial output of P-Net and (e) is the result of unsupervised fine-tuning. (c) and (d) show user-provided scribbles for supervised fine-tuning, and (f) and (g) are their corresponding results.

Fig. 7 shows examples of supervised refinement with additional scribbles. The same initial segmentation and scribbles are used for P-Net + CRF, BIFSeg(-w) and BIFSeg. All these methods improve the segmentation. However, some large mis-segmentations can still be observed for P-Net + CRF and BIFSeg(-w). In contrast, BIFSeg achieves better results with the same set of scribbles. For a quantitative comparison, we measured the segmentation accuracy after a single round of refinement using the same set of scribbles. The result is shown in Table III. BIFSeg achieves significantly better accuracy ($p$-value < 0.05) for the placenta, and previously unseen fetal lungs and maternal kidneys compared with P-Net + CRF and BIFSeg(-w). Fig. 8 shows a visual comparison of unsupervised and supervised fine-tuning of BIFSeg for the same maternal kidney. Table II and Table III show that supervised fine-tuning achieves 3-5 percentage points higher Dice than unsupervised fine-tuning.

**TABLE III:** Quantitative Comparison of P-Net and Three Supervised Refinement Methods With Scribbles for Fetal MRI Segmentation. ${T}_{m}$ is the Machine Time for Refinement. ${\wedge} $ Denotes Previously Unseen Objects. In Each Row, Bold Font Denotes the Best Value. * Denotes ${p}$-Value < 0.05 Compared With the Others

		P-Net	P-Net+CRF	BIFSeg(-w)	BIFSeg
Dice(%)	P	84.57±8.37	88.64±5.84	89.79±4.60	91.93±2.79*
	FB	89.44±6.45	94.04±4.72	95.31±3.39	95.58±1.94
	FL$^{\wedge}$	83.59±6.42	88.92±3.87	89.21±2.95	91.71±3.18*
	MK$^{\wedge}$	85.29±5.08	87.51±4.53	87.78±4.46	89.37±2.31*
$T_{m}$ (s)		-	0.02±0.01*	0.72±0.11	0.74±0.12

P: Placenta, FB: Fetal brain, FL: Fetal lungs, MK: Maternal kidneys.

#### 5) Comparison With Other Interactive Methods:

**Fig. 9.**
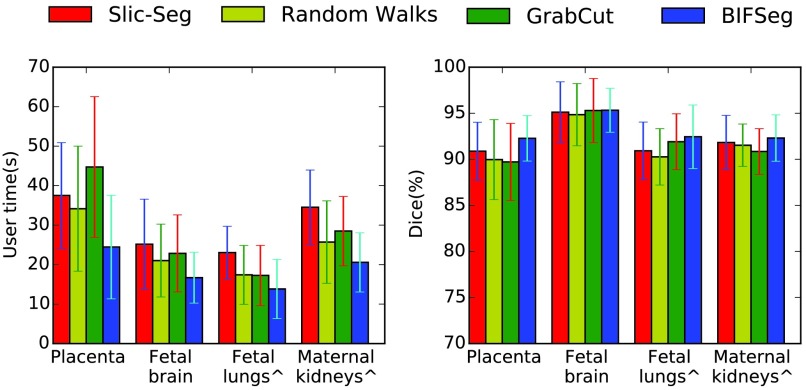
User time and Dice score of different interactive methods for fetal MRI segmentation. $^{\wedge}$ denotes previously unseen objects for BIFSeg.

The two users (an Obstetrician and a Radiologist) used Slic-Seg [21], GrabCut [20], Random Walks [18] and BIFSeg for the fetal MRI segmentation tasks respectively. For each image, the segmentation was refined interactively until it was accepted by the user. The user time and final accuracy of are presented in Fig. 9. It shows that BIFSeg takes noticeably less user time with similar or higher accuracy compared with the other three interactive segmentation methods.

### C. 3D Segmentation of Brain Tumors From T1c and FLAIR

#### 1) Data:

We used the 2015 Brain Tumor Segmentation Challenge (BRATS) training set [31]. The ground truth were manually delineated by experts. This dataset included 274 scans from 198 patients. Each scan used multiple MR sequences with different contrasts. T1c highlights the tumor without peritumoral edema, designated “tumor core” as per [31]. FLAIR highlights the tumor with peritumoral edema, designated “whole tumor” as per [31]. We investigate interactive segmentation of the tumor core from T1c images and the whole tumor from FLAIR images, which is different from previous works on automatic multi-label and multi-modal segmentation [7], [32]. We randomly selected T1c and FLAIR images of 19, 25 patients with a single scan for validation and testing, respectively, and used T1c images of the remaining patients for training. Here the tumor core in T1c images was previously seen while the whole tumor in FLAIR images was previously unseen for the CNNs. All these images had been skull-stripped and resampled to isotropic 1mm^3^ resolution. The maximal side length of bounding boxes of the tumor core and the whole tumor ranged from 40 to 100 voxels, we resized the cropped image region inside a bounding box so that its maximal side length was 80 voxels. Parameter setting was $\lambda = 10.0$, $\sigma = 0.1$, $t_{0} = 0.2$, $t_{1} = 0.6$, $\epsilon = 0.2$, $\omega = 5.0$ based on a grid search with the training data (i.e., whole tumor images were not used for parameter learning).

#### 2) Initial Segmentation Based on PC-Net:

**Fig. 10.**
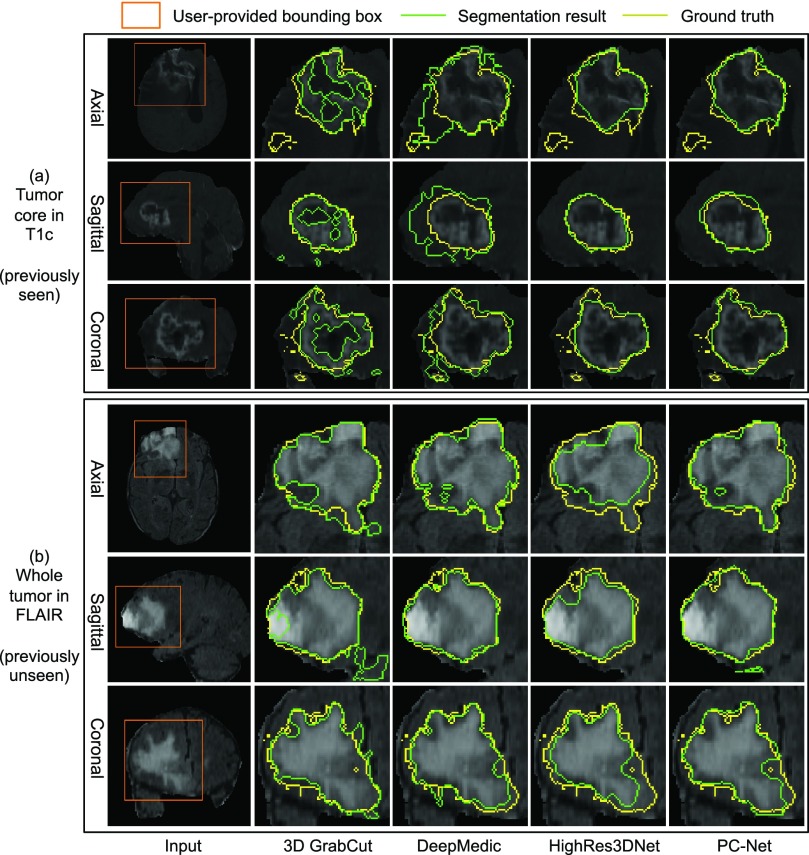
Visual comparison of initial segmentation of brain tumors from a 3D bounding box. The whole tumor in FLAIR is previously unseen in the training set. All these methods use the same bounding box for each test image.

Fig. 10(a) shows an initial result of tumor core segmentation from T1c with a user-provided bounding box. Since the central region of the tumor has a low intensity that is similar to the background, 3D GrabCut obtains large under-segmentations. DeepMedic leads to some over-segmentations. HighRes3DNet and PC-Net obtain similar results, but PC-Net is less complex and has lower memory consumption. Fig. 10(b) shows an initial segmentation result of previously unseen whole tumor from FLAIR. 3D GrabCut fails to get high accuracy due to intensity inconsistency in the tumor region, and the CNNs outperform 3D GrabCut, with DeepMedic and PC-Net performing better than HighRes3DNet. A quantitative comparison is presented in Table IV. It shows that the performance of DeepMedic is low for T1c but high for FLAIR, and that of HighRes3DNet is the opposite. This is because DeepMedic has a small receptive field and tends to rely on local features. It is difficult to use local features to deal with T1c due to its complex appearance but easier to deal with FLAIR since the appearance is less complex. HighRes3DNet has a more complex model and tends to over-fit the tumor core. In contrast, PC-Net achieves a more stable performance on the tumor core and the previously unseen whole tumor. The average machine time for 3D GrabCut, DeepMedic, and PC-Net is 3.87s, 65.31s and 3.83s, respectively (on the laptop), and that for HighRes3DNet is 1.10s (on the cluster).

**TABLE IV:** Dice Score of Initial Segmentation of Brain Tumors From a 3D Bounding Box. All the Methods Use the Same Bounding Box for Each Test Image. ${\wedge}$ Denotes Previously Unseen Objects. In Each Row, Bold Font Denotes the Best Value. * Denotes ${p}$-Value < 0.05 Compared With the Others

	DeepMedic	HighRes3DNet	PC-Net	3D GrabCut
TC	76.68±11.83	83.45±7.87	82.66±7.78	69.24±19.20
WT$^{\wedge}$	84.04±8.50	75.60±8.97	83.52±8.76	78.39±18.66

TC: Tumor core in T1c, WT: Whole tumor in FLAIR.

#### 3) Unsupervised Image-Specific Fine-Tuning:

**Fig. 11.**
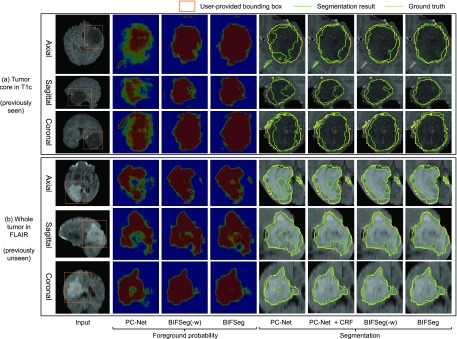
Visual comparison of PC-Net and unsupervised refinement methods without additional scribbles for 3D brain tumor segmentation. The same initial segmentation obtained by PC-Net is used by different refinement methods. (a) Tumor core in T1c (previously seen). (b) Whole tumor in FLAIR (previously unseen).

Fig. 11 shows unsupervised fine-tuning for brain tumor segmentation without additional user interactions. In Fig. 11(a), the tumor core is under-segmented in the initial output of PC-Net. CRF improves the segmentation to some degree, but large areas of under-segmentation still exist. The segmentation result of BIFSeg(-w) is similar to that of CRF. In contrast, BIFSeg performs better than CRF and BIFSeg(-w). A similar situation is observed in Fig. 11(b) for segmentation of previously unseen whole tumor. A quantitative comparison of these methods is shown in Table V. BIFSeg improves the average Dice score from 82.66% to 86.13% for the tumor core, and from 83.52% to 86.29% for the whole tumor.

**TABLE V:** Quantitative Comparison of PC-Net and Unsupervised Refinement Methods Without Additional Scribbles for 3D Brain Tumor Segmentation. ${T}_{m}$ Is the Machine Time for Refinement. ${\wedge}$ Denotes Previously Unseen Objects. In Each Row, Bold Font Denotes the Best Value. * Denotes ${p}$-Value < 0.05 Compared With the Others

		PC-Net	PC-Net+CRF	BIFSeg(-w)	BIFSeg
Dice(%)	TC	82.66±7.78	84.33±7.32	84.67±7.44	86.13±6.86*
	WT$^{\wedge}$	83.52±8.76	83.92±7.33	83.88±8.62	86.29±7.31*
$T_{m}$(s)	TC	-	0.12±0.04*	3.36±0.82	3.32±0.82
	WT$^{\wedge}$	-	0.11±0.05*	3.16±0.89	3.09±0.83

TC: Tumor core in T1c, WT: Whole tumor in FLAIR.

#### 4) Supervised Image-Specific Fine-Tuning:

**Fig. 12.**
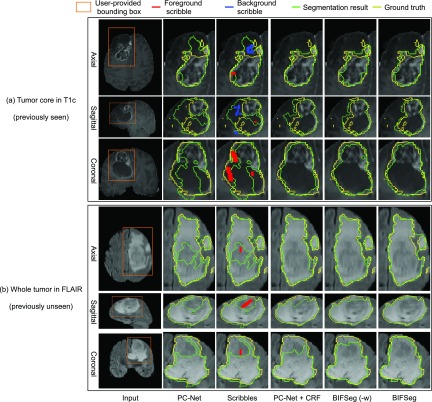
Visual comparison of PC-Net and three supervised refinement methods with scribbles for 3D brain tumor segmentation. The refinement methods use the same initial segmentation and set of scribbles. (a) Tumor core in T1c (previously seen). (b) Whole tumor in FLAIR (previously unseen).

Fig 12 shows refined results of brain tumor segmentation with additional scribbles provided by the user. The same initial segmentation based on PC-Net and the same scribbles are used by CRF, BIFSeg(-w) and BIFSeg. It can be observed that CRF and BIFSeg(-w) correct the initial segmentation moderately. In contrast, BIFSeg achieves better refined results for both the tumor core in T1c and the whole tumor in FLAIR. For a quantitative comparison, we measured the segmentation accuracy after a single round of refinement using the same set of scribbles based on the same initial segmentation. The result is presented in Table VI, showing BIFSeg significantly outperforms CRF and BIFSeg(-w) in terms of Dice. Table V and Table VI show that supervised fine-tuning achieves 1.3-1.8 percentage points higher Dice than unsupervised fine-tuning for brain tumor segmentation.

**TABLE VI:** Quantitative Comparison of PC-Net and Three Supervised Refinement Methods With Additional Scribbles for 3D Brain Tumor Segmentation. ${T}_{m}$ is the Machine Time for Refinement. ${\wedge}$ Denotes Previously Unseen Objects. In Each Row, Bold Font Denotes the Best Value. * Denotes ${p}$-Value < 0.05 Compared With the Others

		PC-Net	PC-Net+CRF	BIFSeg(-w)	BIFSeg
Dice(%)	TC	82.66±7.78	85.93±6.64	85.88±7.53	87.49±6.36*
	WT$^{\wedge}$	83.52±8.76	85.18±6.78	86.54±7.49	88.11±6.09*
$T_{m}$(s)	TC	-	0.14±0.06*	3.33±0.86	4.42±1.88
	WT$^{\wedge}$	-	0.12±0.05*	3.17±0.87	4.01±1.59

TC: Tumor core in T1c, WT: Whole tumor in FLAIR.

#### 5) Comparison With Other Interactive Methods:

**Fig. 13.**
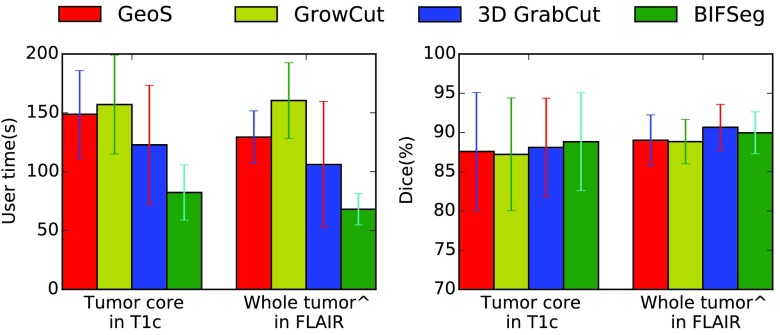
User time and Dice score of different interactive methods for 3D brain tumor segmentation. $^{\wedge}$ denotes previously unseen objects for BIFSeg.

The two users (an Obstetrician and a Radiologist) used GeoS [19], GrowCut [28], 3D GrabCut [29] and BIFSeg for the brain tumor segmentation tasks respectively. The user time and final accuracy of these methods are presented in Fig. 13. It shows that these interactive methods achieve similar final Dice scores for each task. However, BIFSeg takes significantly less user time, which is 82.3s and 68.0s in average for the tumor core and the whole tumor, respectively.

## IV. Discussion and Conclusion

Applying pre-trained models to previously unseen objects is a zero-shot learning problem [33]. While previous works studied zero-shot learning for image classification [34], this paper focused on the context of medical image segmentation. For 2D images, our P-Net was trained with the placenta and fetal brain only, but it performed well on previously unseen fetal lungs and maternal kidneys. There are two main reasons for this. First, these four organs were imaged with the same protocol. They have similar signal to noise ratio and share some common features, such as saliency, contrast and hyper-intensity. Second, compared with FCN and U-Net, P-Net has far fewer parameters without reduction of the receptive field. Therefore, it can generalize better to previously unseen objects. Similarly, the tumor core and whole tumor have some common features, e.g., lower or higher intensity than the remaining brain regions. PC-Net is more compact than HighRes3DNet and less likely to achieve over-fitting, leading to better ability to deal with the unseen whole tumor. Table IV shows that DeepMedic achieves higher accuracy for the whole tumor. The reason is that our 3D experiment tends to learn to recognize hyper-intensity regions where local features have a higher influence than global features, and the relatively smaller receptive field of DeepMedic is more suitable for this task compared with the PC-Net and HighRes3DNet. However, DeepMedic has a lower performance when dealing with the tumor core in T1c images where the intensity is more inhomogeneous.

Our BIFSeg framework is theoretically applicable to different CNN models. However, this research focuses on interactive segmentation, where short inference time and memory efficiency of the network are key requirements to enable responsive user interfaces and to work on machines with limited GPU resources. This is especially critical for 3D image segmentation. DeepMedic takes over 60 seconds for inference, while HighRes3DNet has too large a memory consumption to work on a laptop. They are thus less suitable for interactive segmentation compared with PC-Net. We have designed PC-Net with the explicit requirement of interactive runtime on a laptop. To ensure that PC-Net was suitable for the brain tumor segmentation task despite the gain in efficiency, we compared the initial fully automated output of PC-Net, DeepMedic and HighRes3D. Then, we only used PC-Net for the interactive segmentation pipeline of BIFSeg.

Dealing with unseen objects is a major advantage compared with traditional CNNs and even transfer learning [25] or weakly supervised learning [3], since for some objects it does not require annotated instances for training at all. It therefore reduces the efforts needed for gathering and annotating training data and can be applied to some unseen organs directly. In this paper we only used at most two objects in the training set. To further increase BIFSeg’s ability to generalize, it is of interest to use a larger training set with more patients, organs and image modalities, since a large training set with a wide variety of different image contexts helps to learn common features among different objects [35].

Our proposed framework accepts bounding boxes and optional scribbles as user interactions. Bounding boxes in test images are provided by the user, but they could potentially be obtained by automatic detection [36] to further increase efficiency. Experimental results show that the image-specific fine-tuning improves the segmentation performance. This acts as a post-processing step after the initial segmentation and outperforms CRF. Though unsupervised fine-tuning helps to correct small mis-segmentations when the initial fully-automated performance is satisfactory, it may lead to under-performance when dealing with some complex cases, considering the distribution mismatch between the training and testing data. To address this problem, BIFSeg allows optional supervised fine-tuning that leverages user interactions to achieve higher robustness and accuracy. Since the scribbles are provided only in mis-segmented areas, the variations of position and length of scribbles are limited and much smaller than that of freely drawn scribbles used in traditional methods such as Random Walks [18] and Slic-Seg [21], and the output of BIFSeg also varies slightly with varying scribbles, as shown in Fig. 8. We found that taking advantage of uncertainty plays an important role for the image-specific fine-tuning process. The uncertainty is defined based on softmax probability and geodesic distance to scribbles if scribbles are given. Previous works [37] suggest that test-time dropout also provides classification uncertainty. However, test-time dropout is less suited for interactive segmentation since it leads to longer computational time. In our experiments, hyper-parameters of BIFSeg (e.g., $\lambda $) were fixed globally in the testing stage. Using object-specific parameter adjustment or allowing the user to tune these parameters for each test image in the interactive procedure may help to get better segmentation accuracy.

In conclusion, we propose an efficient deep learning-based framework for interactive 2D/3D medical image segmentation. It uses a bounding box-based CNN for binary segmentation and can segment previously unseen objects. A unified framework is proposed for both unsupervised and supervised refinements of the initial segmentation, where image-specific fine-tuning based on a weighted loss function is proposed. Experiments on segmenting multiple organs from 2D fetal MRI and brain tumors from 3D MRI show that our method performs well on previously unseen objects and the image-specific fine-tuning outperforms CRF. BIFSeg achieves similar or higher accuracy with fewer user interactions and less user time than traditional interactive segmentation methods.
